# Identification of victims of the collapse of a mine tailing dam in Brumadinho

**DOI:** 10.1080/20961790.2022.2113623

**Published:** 2023-02-12

**Authors:** Ricardo Moreira Araújo, Yara Vieira Lemos, Erlon Dias do Nascimento, Anna Helena Silva Paraizo, Alberto Julius Alves Wainstein, Ana Paula Drummond-Lage

**Affiliations:** a Faculty of Medical Sciences of Minas Gerais, Belo Horizonte, Brazil; b Instituto Médico Legal André Roquette, Belo Horizonte, Brazil; c Corpo de Bombeiros Militar de Minas Gerais, Juiz de Fora, Brazil

**Keywords:** Disasters, victim identification, tailings, mining, legal medicine, developing countries, man-made disasters, traumatic amputation

## Abstract

The collapse of the B1 Dam of VALE SA mining company in Brumadinho, Minas Gerais, Brazil was the largest humanitarian disaster and occupational accident in the country’s history, and it posed challenges regarding the management and identification of multiple victims. We evaluated the impact of the iron ore tailings on the victims’ bodies. We examined the scientific identification of the victims and the dynamics of the disaster over the 1st year after it occurred. We also determined the socio-demographic profiles of the victims. In this retrospective, cross-sectional study, we investigated the expert reports of the victims’ biological remains from 25 January 2019 to 25 January 2020. We analysed the socio-demographic data, identification methods, identification status, identification time, and necroscopic information. During the study period, 259 of 270 victims were identified, and 603 biological materials were analysed; among them, 86.2% were body parts and 13.8% were whole bodies. Of the total cases registered that year, 476 (78.9%) were submitted during the first 10 weeks after the disaster. Friction ridge analysis accounted for 67.9% of primary identifications and DNA analysis did so for 91.6% of re-identification cases. Body dismemberment was 3.4 times greater among mine workers than among community victims. Adult males accounted for the greatest number of victims (*P* < 0.001). Polytraumatic injury was the prevalent single cause of death. Necropsy examination revealed the occurrence of asphyxia in 7% of cases. The higher number of fatalities and greater dismemberment among employees than with community residents underlines the occupational dangers in the mining industry and clarifies the dynamics of the disaster. In the initial weeks after the dam collapsed, friction ridge analysis was the most appropriate method for identification. Subsequently, DNA analysis became the most-used technique for identification and re-identification owing to the great volume of body parts and decomposed biological tissue. Autopsy allowed diagnosis of the causes of death to be clarified according to the Brazilian criminal legal system.

## Key points

The collapse of B1 Dam of VALE SA mining company in Brumadinho was the greatest humanitarian disaster and occupational accident in the country’s history.

This article examined the challenges and solutions regarding the management and identification of the disaster’s many victims.

Of the victims, 95.9% were identified during the 1st year after the disaster; 78.9% were identified in the first 10 weeks.

Polytraumatic injury accounted for 86.2% of deaths; it was observed more frequently among mine employees than among community residents.

In the first weeks after the disaster, friction ridge analysis was mostly used for identification; subsequently, DNA analysis was employed for most identification and re-identification cases.

## Introduction

**Figure 1.**
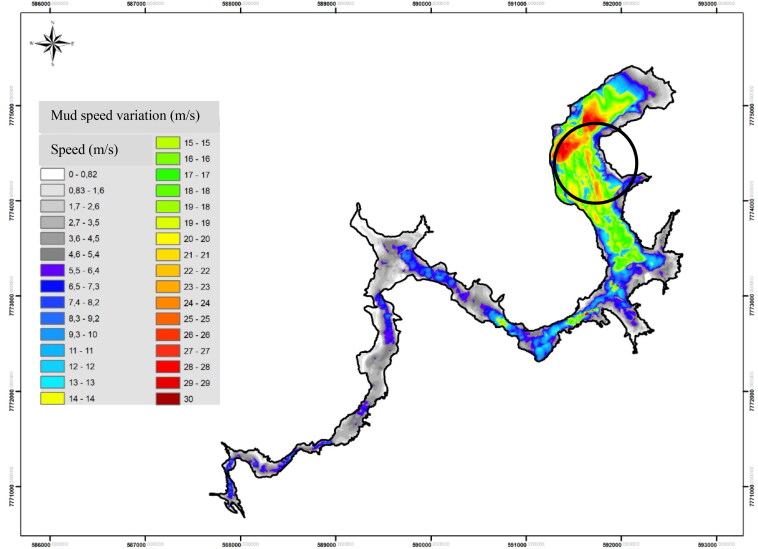
Simulation of mass tailing behaviour. Velocity of the iron ore tailing mass in its path from the B1 Dam to the Paraopeba River in 2019. Mining company building area is shown in the circle (Fire Department file, Minas Gerais, Brazil, 2019, reproduced with permission).

On 25 January 2019, the collapse of a deactivated iron ore tailings dam raised upstream, located in the Córrego do Feijão community in Brumadinho, Minas Gerais, Brazil, released around 1.3 × 10^7^ m^3^ of tailings on mine workers and local residents. The damage extended beyond the mining complex [1–4]. The mechano-kinetic energy of the tailings dragged along large, heavy machinery as well as trees, animals, and people. The waste left in its path was 18 m deep at some points, and its surface area measured 2.7 km^2^ (Figure 1) [3–5].

Through the scope of forensics, the focus on human identification in response to catastrophic events is highlighted by the term disaster victim identification (DVI), applied by the International Criminal Police Organization (INTERPOL) in its manual on the topic [6–10]. That emphasis does not minimise the necessity of concomitantly conducting other steps in the criminal investigation: confirmation of death; determination of cause of death; and determination of aggravating or qualifying circumstances that played a part in the casualties [11].

The energy of the mass of tailings and destructive action on victims, the dimensions of the mass, the temporal extension of its force, the search efforts, and decomposition posed challenges regarding the management of the Brumadinho DVI, which was conducted by the Medical Examiner’s Office André Roquette (IMLARBH) [12–22]. It was considered the largest humanitarian disaster and greatest occupational accident in Brazil’s history. Thus, the task force responsible for the search, localisation, and identification of victims turned out to be the world’s largest search operation [2, 4, 23].

The present study examined the Brumadinho DVI towards attaining a better understanding of the disaster response [24]. Using distinct ­postmortem intervals, it presents the victims’ socio-demographic profiles, analyses the destructive action of the tailings on humans, evaluates the injuries and causes of death, and assesses the methods of human identification among the types of found remains.

## Materials and methods

In this retrospective, cross-sectional study, we evaluated the official forensic reports (Medical Examiner, Odontological, Anthropological, Friction ridges, and DNA Molecular Biology/technology) of all biological remains found in the search operation of the B1 Dam collapse of the Córrego do Feijão Mine provided by IMLARBH from 25 January 2019 to 25 January 2020. We excluded from this study cases forensically defined as non-human.

We organised and categorised the data in a single Excel spreadsheet. In creating the database, we redacted the original numbers of the identified victims’ reports and substituted them with serial numbers to ensure privacy. Re-identification was conducted by following the same criteria to allow primary identification of a victim.

### Socio- demographic details of identified victims

Among 751 cases assessed, we excluded 148 as non-human; thus, the final sample comprised 603 cases. We obtained the following variables: victim’s age; sex; and whether they were service providers or employees of VALE SA, a private company ranking among the largest miners in the world, or community members.

The study was approved by the IMLARBH’s Study Centre and the Research Ethics Committee of the Faculty of Medical Sciences of Minas Gerais (Approval Number 3.487.250).

### Identification methods

We categorised the methods as follows: fingerprints, odontology, anthropology, and DNA. We recognised three types of status: identification (primary identification of biological remains of an unidentified victim); re-identification (identification of biological remains belonging to a previously identified victim); and inconclusive (cases where after exhaustive trials for DNA extraction it was impossible to conclude whether the remains were human).

### Identification time

We categorised the response time variable as follows: time of localisation (time of filing of case at the IMLARBH); time of identification (time of conclusion of official identification report); time of discovery (time elapsed, in days, between the time of disaster and time of discovery of the case by search teams); and time of identity confirmation (time elapsed, in days, between the time of localisation and time of identification).

### Medical examiner

Data were collected from official reports; they included the cause of death and body integrity and fragmentation. The body was considered whole if it presented no loss or avulsion of organs, segments, or parts. In more advanced stages of decomposition, the body was considered whole even if skeletonised, liquefactive, or saponified as long as at least 90% of the bone structure remained. A body part was defined as any biological material that could not be classified as a whole body but that conformed with the anatomical definition of its original body region. Head and torso parts were classified as whole when over 90%, respectively, of the cranium and torso-abdomen bone structures were present. With superior limbs, the parts were defined as whole when their three composing subdivisions (arm, forearm, and hand) presented a complete bone structure, or when one of the subdivisions had a maximum loss of 50%. The definition of wholeness of a limb did not reflect symmetry or the presence of both limbs. The same criteria were applied to the inferior limbs. We defined biological material as non-classifiable biological tissue or a body part with loss of anatomical parameters (macro- or microscopic) whose original bodily location could be identified. Thus, we included as biological material fragments of soft tissue (shreds of skin, entrails, muscle, and scalp) as well as small bone fragments.

### Statistical analysis

We submitted the quantitative variables to the Shapiro-Wilk normality test. We assessed the association between the qualitative variables using binary logistic models and Pearson’s chi-square and Fisher’s exact tests. To compare quantitative variables among three or more groups, we applied the Kruskal-Wallis test followed by a *post hoc* Dunn test in the case of significance. We conducted the analyses using free software R version 4.0.2 (R Foundation) and *P* < 0.05 was considered significant.

## Results

Therefore, the final sample comprised 603 cases, corresponding to 259 bodies: 206 (79.5%) were male. Of the final sample, 95.8% were adults (aged 20–59 years) according to the age rating of the Brazilian Institute of Geography and Statistics. Among the victims, five were young (aged 0–19 years) and six were elderly (aged 60 years or older). Among 238 deceased workers, 122 (51.3%) were employed by VALE SA and 116 (48.7%) were third-party employees (Table 1).

**Table 1.** Socio-demographic details of the 259 Brumadinho disaster victims.

Variables	*N* (%)^a^
Sex	
Female	53 (20.5)
Male	206 (79.5)
Age rating IBGE^b^	
0–4	1 (0.4)
10–14	2 (0.8)
15–19	2 (0.8)
20–24	11 (4.2)
25–29	28 (10.8)
30–34	51 (19.7)
35–39	61 (23.6)
40–44	38 (14.7)
45–49	30 (11.6)
50–54	17 (6.6)
55–59	12 (4.6)
60–64	3 (1.2)
65–69	2 (0.8)
75–79	1 (0.4)
Category	
Community	21 (8.1)
Worker	238 (91.9)

^a^Percentages may not total 100 due to rounding.

^b^IBGE: Brazilian Institute of Geography and Statistics.

Most cases (520, 86.2%) consisted of body parts. Whole bodies were found in 83 (13.8%) cases, 17 of which belonged to the 21 community victims (81.0%). Among the 238 workers (VALE SA and third-party employees), the whole body was found in 66 (27.7%) cases. The most commonly found body part was an incomplete inferior limb (101, 16.8%) followed by biological tissue (75, 12.4%). An incomplete inferior limb was mostly found among workers (*P* <0.001; Table 2). Body dismemberment was three times more evident among workers than with with community victims.

**Table 2.** Most frequently found body parts.

Variables	Inc. IL (*n*, %)	Whole body (*n*, %)	Biological tissue (*n*, %)	Inc. torso, comp. IL (*n*, %)	Inc. torso, inc. IL (*n*, %)	Inc. SL (*n*, %)	Inc. torso (*n*, %)
Sex	*P* = 0.017^a^	*P* = 0.803^a^	*P* = 0.450^a^	*P* = 1.000^a^	*P* = 0.433^a^	*P* = 0.469^a^	*P* = 0.808^a^
Female	11 (9.1)	18 (14.9)	18 (14.9)	8 (6.6)	10 (8.3)	9 (7.4)	5 (4.1)
Male	90 (18.7)	65 (13.5)	57 (11.8)	31 (6.4)	28 (5.8)	28 (5.8)	25 (5.2)
Age (years)	*P* = 0.814	*P* < 0.001^a^	*P* = 0.469^a^	*P* = 0.763^b^	*P* = 0.980^b^	*P* = 0.939^b^	*P* = 0.009^a^
Up to 18 years	–	4 (100.0)	–	–	–	–	–
19–29	17 (15.5)	13 (11.8)	11 (10.0)	7 (6.4)	7 (6.4)	8 (7.3)	7 (6.4)
30–39	36 (16.1)	38 (17.0)	28 (12.6)	12 (5.4)	14 (6.3)	13 (5.8)	4 (1.8)
40–49	31 (17.6)	17 (9.7)	20 (11.4)	11 (6.2)	10 (5.7)	11 (6.2)	17 (9.7)
50–59	16 (20.3)	7 (8.9)	15 (19.0)	8 (10.1)	6 (7.6)	5 (6.3)	2 (2.5)
60–79	1 (9.1)	4 (36.4)	1 (9.1)	1 (9.1)	1 (9.1)	–	–
Category 1	*P* = 0.063^a^	*P* < 0.001^a^	*P* = 0.215^a^	*P* = 0.335^a^	*P* = 0.303^a^	*P* = 0.877^a^	*P* = 0.260^a^
Community	–	17 (68.0)	1 (4.0)	–	–	1 (4.0)	1 (4.0)
Third Party	52 (18.3)	36 (12.7)	41 (14.4)	21 (7.4)	21 (7.4)	17 (6.0)	10 (3.5)
VALE SA	49 (16.7)	30 (10.2)	33 (11.2)	18 (6.1)	17 (5.8)	19 (6.5)	19 (6.5)
Category 2	*P* < 0.001^a^	*P* < 0.001^a^	*P* = 0.042^a^	*P* = 0.063^c^	*P* = 0.063^c^	*P* = 0.348^c^	*P* = 0.287^c^
Community	–	17 (35.4)	1 (2.1)	–	–	1 (2.1)	4 (8.3)
Worker	101 (18.2)	66 (11.9)	74 (13.3)	39 (7.0)	38 (6.8)	36 (6.5)	26 (4.7)
**Total**	**101**	**83**	**75**	**39**	**38**	**37**	**30**

The proportions were calculated by line. Comp.: complete; inc.: incomplete; SL: superior limb; IL: inferior limb. ^a^ Chi-square test; ^b^ binary logistic model; ^c^ Fisher’s exact test.

Responsible for 574 (95.2%) cases, polytraumatic injury (PTI) was the main single cause of death. Other single causes of death were observed in seven cases (1.2%): asphyxia (4, 0.7%); spinal cord injury (1, 0.2%); and cranioencephalic trauma (2, 0.3%). There was an association between asphyxia and the other 22 (3.7%) causes of death: PTI and asphyxia (19, 3.2%); cranioencephalic trauma and asphyxia (2, 0.3%); and torso trauma and asphyxia (1, 0.2%). With respect to body parts, a complete torso, complete superior limb, and incomplete inferior limb with PTI plus asphyxia (*P* = 0.025) was the most common followed by a complete head, incomplete torso, and complete superior limb (*P* < 0.001; Table 3).

**Table 3.** Causes of death in the Brumadinho disaster.

Body part	Total (*n*, %)	Asphyxia (*n*, %)	PTI (*n*, %)	PTI + asphyxia (*n*, %)	TCEC (*n*, %)	TCEC + asphyxia (*n*, %)	TRMC (*n*, %)	TTC + asphyxia (*n*, %)	*P*-value
Whole body	83 (13.8)	4 (100)	66 (11.5)	7 (36.8)	2 (100)	2 (100)	1 (100)	1 (100)	<0.001
Inc. head, inc. torso, comp. SL	24 (4.0)	–	23 (4.0)	1 (5.3)	–	–	–	–	0.998
Comp. head, inc. torso, inc. SL	3 (0.5)	–	3 (0.5)	–	–	–	–	–	–
Inc. torso, comp. SL	6 (1.0)	–	6 (1.0)	–	–	–	–	–	–
Inc. torso, inc. IL	38 (6.3)	–	38 (6.6)	–	–	–	–	–	–
Inc. head, inc. torso, inc. SL, inc. IL	3 (0.5)	–	3 (0.5)	–	–	–	–	–	–
Comp. head, comp. torso, inc. SL, inc. IL	2 (0.3)	–	2 (0.3)	–	–	–	–	–	–
Inc. head, comp. torso, inc. SL, inc. IL	2 (0.3)	–	2 (0.3)	–	–	–	–	–	–
Comp. torso, inc. SL, inc. IL	2 (0.3)	–	2 (0.3)	–	–	–	–	–	–
Inc. torso, comp. SL, inc. IL	5 (0.8)	–	4 (0.7)	1 (5.3)	–	–	–	–	0.579
Comp. torso, comp. SL, inc. IL	2 (0.3)	–	1 (0.2)	1 (5.3)	–	–	–	–	0.025
Comp. head, comp. torso, comp. SL, comp. IL, body almost whole	17 (2.8)	–	17 (3.0)	–	–	–	–	–	–
Inc. torso, comp. IL	39 (6.5)	–	39 (6.8)	–	–	–	–	–	–
Comp. torso, inc. SL, comp. IL	1 (0.2)	–	1 (0.2)	–	–	–	–	–	–
Inc. torso, comp. SL, comp. IL	7 (1.2)	–	6 (1.0)	1 (5.3)	–	–	–	–	0.815
Comp. torso, comp. SL, comp. IL	24 (4.0)	–	22 (3.8)	2 (10.5)	–	–	–	–	0.861
Inc. torso, inc. SL	10 (1.7)	–	10 (1.7)	–	–	–	–	–	–
Inc. head, inc. torso	3 (0.5)	–	3 (0.5)	–	–	–	–	–	–
Comp. head, inc. torso	1 (0.2)	–	1 (0.2)	–	–	–	–	–	–
Comp. head, comp. torso	1 (0.2)	–	–	1 (5.3)	–	–	–	–	–
Comp. head, inc. SL	3 (0.5)	–	3 (0.5)	–	–	–	–	–	–
Comp. head, inc. torso, comp. SL	12 (2.0)	–	8 (1.4)	4 (21.1)	–	–	–	–	<0.001
Comp. head, comp. torso, comp. SL	5 (0.8)	–	4 (0.7)	1 (5.3)	–	–	–	–	0.579
Inc. head, inc. torso, inc. SL	9 (1.5)	–	9 (1.6)	–	–	–	–	–	–
Inc. head, comp. torso, comp. SL	3 (0.5)	–	3 (0.5)	–	–	–	–	–	–
Only biological tissue	75 (12.4)	–	75 (13.1)	–	–	–	–	–	–
Only inc. SL	37 (6.1)	–	37 (6.4)	–	–	–	–	–	–
Only comp. SL	18 (3.0)	–	18 (3.1)	–	–	–	–	–	–
Only inc. IL	101 (16.8)	–	101 (17.6)	–	–	–	–	–	–
Only comp. IL	16 (2.7)	–	16 (2.8)	–	–	–	–	–	–
Only comp. torso	30 (5.0)	–	30 (5.2)	–	–	–	–	–	–
Only inc. head	18 (3.0)	–	18 (3.1)	–	–	–	–	–	–
Only comp. head	3 (0.5)	–	3 (0.5)	–	–	–	–	–	–
**Total**	**603**	**4**	**574**	**19**	**2**	**2**	**1**	**1**	–

The *P*-values refer to the binary logistic model. Comp.: complete; inc.: incomplete; SL: superior limb; IL: inferior limb; PTI: polytraumatic injury; TCEC: spinal cord injury; TRMC: cranioencephalic trauma; TTC: torso trauma.

Each victim was identified using at least one method: with 259 (43.0%) cases, it was primary identification; with 344 (57.0%), it was re-identification. Regarding grouped identification and re-identification of the 603 cases, DNA analysis was applied in 357 (59.2%), friction ridge analysis in 193 (32.0%), odontology in 31 (5.1%), and anthropology in five (0.8%). Combined identification methods were used in 17 (2.8%) cases. With respect to identification methods, anthropology was more frequently employed than DNA analysis with whole bodies (*P* < 0.001); with an incomplete head, incomplete torso, and incomplete superior limbs, anthropology was more commonly used than friction ridge analysis (*P* < 0.001; Table 4).

**Table 4.** Identification methods used for body parts of the Brumadinho victims.

Body part	Anthropology	DNA	Associated methods	Odontology	Friction ridges	*P*-value
Whole body	1 (20)	1 (0.3)	7 (41.2)	4 (12.9)	70 (36.3)	<0.001*
Inc. head, inc. torso, comp. SL	–	1 (0.3)	1 (5.9)	3 (9.7)	19 (9.8)	0.990
Comp. head, inc. torso, inc. SL	–	–	–	2 (6.5)	1 (0.5)	0.999
Inc. torso, comp. SL	–	2 (0.6)	–	–	4 (2.1)	0.481
Inc. torso, inc. IL	–	38 (10.6)	–	–	–	–
Inc. head, inc. torso, inc. SL, inc. IL	–	2 (0.6)	–	1 (3.2)	–	0.218
Comp. head, comp. torso, inc. SL, inc. IL	–	–	–	2 (6.5)	–	–
Inc. head, comp. torso, inc. SL, inc. IL	–	–	–	2 (6.5)	–	–
Comp. torso, inc. SL, inc. IL	–	1 (0.3)	–	–	1 (0.5)	0.982
Inc. torso, comp. SL, inc. IL	–	–	–	–	5 (2.6)	–
Comp. torso, comp. SL, inc. IL	–	–	–	–	2 (1)	–
Comp. head, comp. torso, comp. SL, comp. IL, body almost whole	–	–	3 (17.6)	–	14 (7.3)	0.999
Inc. torso, comp. IL	1 (20)	38 (10.6)	–	–	–	0.512
Comp. torso, inc. SL, comp. IL	–	–	–	1 (3.2)	–	–
Inc. torso, comp. SL, comp. IL	–	–	2 (11.8)	–	5 (2.6)	0.999
Comp. torso, comp. SL, comp. IL	1 (20)	–	–	1 (3.2)	22 (11.4)	0.182
Inc. torso, inc. SL	–	10 (2.8)	–	–	–	–
Inc. head, inc. torso	–	1 (0.3)	1 (5.9)	1 (3.2)	–	0.999
Comp. head, inc. torso	–	–	–	–	1 (0.5)	–
Comp. head, comp. torso	–	–	–	–	1 (0.5)	–
Comp. head, inc. SL	–	–	–	1 (3.2)	2 (1.0)	0.104
Comp. head, inc. torso, comp. SL	–	1 (0.3)	–	–	11 (5.7)	0.997
Comp. head, comp. torso, comp. SL	–	–	–	1 (3.2)	4 (2.1)	0.999
Inc. head, inc. torso, inc. SL	1 (20)	3 (0.8)	1 (5.9)	3 (9.7)	1 (0.5)	<0.001*
Inc. head, comp. torso, comp. SL	–	–	1 (5.9)	–	2 (1.0)	0.999
Only biological tissue	–	75 (21.0)	–	–	–	–
Only inc. SL	–	21 (5.9)	–	–	16 (8.3)	0.282
Only comp. SL	–	6 (1.7)	–	–	12 (6.2)	0.997
Only inc. IL	1 (20)	100 (28)	–	–	–	0.694
Only comp. IL	–	16 (4.5)	–	–	–	–
Only comp. torso	–	30 (8.4)	–	–	–	–
Only inc. head	–	10 (2.8)	1 (5.9)	7 (22.6)	–	0.998
Only comp. head	–	1 (0.3)	–	2 (6.5)	–	0.999
**Total**	**5**	**357**	**17**	**31**	**193**	–

The *P*-values refer to the binary logistic model. Comp.: complete; inc.: incomplete; SL: superior limb; IL: inferior limb.

* Significant differences.

Friction ridge analysis was applied as a single method in 176 (68.0%) primary identifications; it was followed by DNA analysis in 42 (16.2%) identifications. Odontology was employed in 23 cases and anthropology in two cases, accounting for 25 (9.7%) identifications. Combined methods were utilised in 16 (6.2%) identifications.

With re-identification, DNA analysis was used in 315 (91.6%) cases; it was followed by friction ridge analysis (17, 4.9%) and odontology and anthropology (11, 3.2%). Combined methods were employed in one re-identification.

During the first 10 weeks of the response to the Brumadinho disaster, 476 (78.9%) of the 603 cases were reported to the IMLARBH. Among the 476, 138 (29.0%) were reported in the 1st week of response. There were 211 in February and 127 in March, respectively. After April (28 cases), the number of cases reported did not attain triple figures. This decreasing tendency persisted in subsequent months.

The general average time of localisation was (58.1 ± 85.8) d. The longest average time of localisation was for the following parts: complete head, complete torso, interconnected incomplete superior limb, and incomplete inferior limb (over 214 d); that was followed by complete head, incomplete torso, and incomplete superior limb (over 172 d). The longest average time for identity confirmation was for materials defined as biological tissue (over 80 d), an incomplete head (over 77 d), and complete inferior limb (over 76 d; Table 5).

**Table 5.** Time of localisation and identification of body parts.

Body part	Time of localisation (d)	Time of identification (d)
Whole body	13.0 ± 17.6 (6.0)	1.5 ± 3.1 (1.0)
Inc. head, inc. torso, comp. SL	24.0 ± 37.0 (8.0)	3.2 ± 12.2 (0.5)
Comp. head, inc. torso, inc. SL	172.7 ± 155.3 (217.0)	0.7 ± 1.2 (0.0)
Inc. torso, comp. SL	31.5 ± 26.4 (30.0)	14.3 ± 22.1 (1.5)
Inc. torso, inc. IL	53.5 ± 82.4 (27.5)	73.1 ± 58.3 (49.5)
Inc. head, inc. torso, inc. SL, inc. IL	72.7 ± 85.3 (50)	16.3 ± 25.7 (2.0)
Comp. head, comp. torso, inc. SL, inc. IL	214.5 ± 119.5 (214.5)	0.0*
Inc. head, comp. torso, inc. SL, inc. IL	129.5 ± 166.2 (129.5)	1.5 ± 2.1 (1.5)
Comp. torso, inc. SL, inc. IL	39.5 ± 40.3 (39.5)	29.0 ± 39.6 (29.0)
Inc. torso, comp. SL, inc. IL	3.8 ± 4.4 (1.0)	1.0*
Comp. torso, comp. SL, inc. IL	7.5 ± 5.0 (7.5)	1.0*
Comp. head, comp. torso, comp. SL, comp. IL, body almost whole	35.9 ± 72.2 (5.0)	1.9 ± 5.1 (1.0)
Inc. torso, comp. IL	36.4 ± 58.4 (15.0)	72.3 ± 60.8 (53.0)
Comp. torso, inc. SL, comp. IL	252*	0*
Inc. torso, comp. SL, comp. IL	11.3 ± 10.8 (8.0)	0.6 ± 0.8 (0.0)
Comp. torso, comp. SL, comp. IL	20.1 ± 28.7 (10.0)	0.6 ± 0.7 (0.5)
Inc. torso, inc. SL	52.9 ± 83.7 (28.0)	67.5 ± 31.8 (66.5)
Inc. head, inc. torso	82.3 ± 102.1 (39.0)	35.7 ± 60.0 (1.0)
Comp. head, inc. torso	16*	3*
Comp. head, comp. torso	3*	1*
Comp. head, inc. SL	12.3 ± 0.6 (12.0)	0.7 ± 0.6 (1.0)
Comp. head, inc. torso, comp. SL	12.6 ± 17.7 (4.0)	4.5 ± 13.4 (1.0)
Comp. head, comp. torso, comp. SL	12.2 ± 16.7 (5.0)	1.0 ± 1.2 (1.0)
Inc. head, inc. torso, inc. SL	32.9 ± 49.0 (19.0)	33.8 ± 62.7 (2.0)
Inc. head, comp. torso, comp. SL	11.3 ± 11.0 (6.0)	1.0 ± 1.0 (1.0)
Only biological tissue	63.8 ± 71.5 (48.0)	80.4 ± 38.3 (77.0)
Only inc. SL	115.9 ± 126.7 (50)	34.5 ± 33.7 (29.0)
Only comp. SL	29.0 ± 24.7 (18.5)	34.1 ± 56.5 (1.0)
Only inc. IL	118.0 ± 113.8 (57.0)	67.2 ± 58.8 (50.0)
Only comp. IL	17.1 ± 18.0 (8.5)	76.7 ± 75.2 (43.5)
Only complete torso	54.4 ± 44.8 (51.5)	77.4 ± 51.4 (70.0)
Only incomplete head	108.9 ± 124.6 (54.0)	36.9 ± 32.2 (29.5)
Only complete head	15.7 ± 3.5 (16.0)	25.3 ± 39.6 (3.0)
**Total**	**58.1 ± 85.8 (24.0)**	**43.45 ± 53.08 (29.0)**

* only one case; comp.: complete; inc.: incomplete; SL: superior limb; IL: inferior limb.

**Figure 2.**
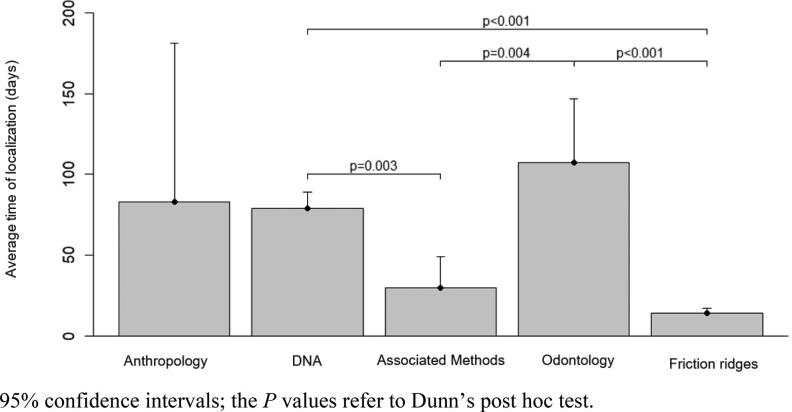
Average time of localisation and identification according to forensic methods (95% confidence intervals); the *P*-values refer to Dunn’s *post hoc* test.

In the 10 weeks following the disaster, 217 (80.4%) of the 259 victims listed by the authorities were identified. In January, 88 (32.6%) victims were identified; there were 101 (37.4%) in February and 28 (10.4%) in March. Regarding identification methods, the longest times of localisation were through odontology and anthropology; the shortest was for friction ridge analysis and combined methods (Figure 2).

## Discussion

Most of the cases registered with the IMLARBH were concentrated in the first 24 d following the disaster. During that period, 301 remains (bodies and parts) were obtained from the search task force located in Brumadinho: they accounted for 49.9% of the cases registered in the 1st year after the disaster. The considerable number of cases registered in a brief period overwhelmed the response capacity of forensics services for DVI [6, 25]. With the time of localisation of 58.1 d, the IMLARBH dealt with 462 cases, which amounted to 76.6% of all the cases registered in the 1st year of response.

The Brumadinho disaster caused 259 fatalities, and there were 603 cases of victims’ body parts. The degree of dismemberment posed great challenges to the task force and required the need to apply higher-cost identification techniques, such as DNA analysis [26, 27]. During the first 24 days after the disaster, 167 primary identifications (64.5%) were made. After 58 d, 214 (82.6%) victims had been primarily identified—mostly (170, 79.4%) by friction ridge analysis.

Brazil operates a civil database called Ante Mortem (AM) for friction ridge analysis; that was used in conjunction with Alethia System, which was developed by the Federal Police of Brazil [9]. Alethia System is a biometric scanner connected to a portable Automated Fingerprint Identification System (AFIS). This combination added agility and promptness in the DVI. The substantial number of cases in the first weeks after the disaster and subsequent decomposition supported using friction ridge analysis as the primary forensic method. That method allows greater accuracy during less advanced stages of putrefaction; it has been applied with other DVIs in Brazil and other countries and yielded different results according to the availability of a database and putrefaction conditions [9, 28]. The availability of civil registers on individual dactyloscopy is highly important for friction ridge analysis.

During a landslide in a mountainous area of Rio de Janeiro in 2011, friction ridge analysis was used to make 54.4% of identifications [8]. In the LaMia Flight 2933 tragedy in 2016 in Colombia, it was employed for 90.1% of identifications [9]. However, in countries lacking a governmental database, friction ridge analysis may produce less impressive results: that was the case in DVI following a terrorist attack in Nice on 14 July 2016, where only 21% of victims could be identified using fingerprints.

In Brumadinho, following the period of intense registration of cases during the first 58 d, 45 (17.4%) victims were primarily identified over the next 307 d. In all, 259 identifications were made during the 1-year period of the present study. The identification methods used for those 45 cases were as follows: DNA analysis (62.0%); odontology (22.0%); friction ridge analysis (15.5%); combined methods (6.5%); and anthropology (2.2%).

The use of those methods is justified by the gradual progression of the effects of decomposition (and resulting loss of soft tissue of the digital pulp) and the high degree of dismemberment (Supplementary Table 1). Friction ridge analysis soon begins to decline in effectiveness for primary identification; however, that analysis should be prioritised owing to its low cost and swift applicability. The presence of adipocere and use of friction ridge techniques [29] allowed six primary identifications to be made during the 4th month and one primary identification in the 10th month (268 d after the disaster). Owing to the high degree of dismemberment and ongoing effects of putrefaction, there was a marked increase in identification employing DNA analysis after the 2nd month of the disaster (62.0% primary identification, 91.6% re-identification). In the 10 months after the disaster, 350 identification reports were made using DNA analysis.

Creating a database of parental genetic profiles in the first 2 weeks following the disaster contributed to the above results. Relatives cooperated by providing biological samples to build the database, which positively affected the results obtained with DNA analysis. This finding is in line with those related to the September 11 attacks in the USA in 2001, which underline the importance of applying DNA analysis of dismemberment or fragmentation [30].

In Brumadinho, forensic odontology was responsible for 9.2% of primary identifications. The durability of bone and teeth allows them to be used for DVI for over 12 months after an incident. The odontological AM database depends on the quality of the previous radiographic registry, which is one of the main limiting factors for applying odontology [31]. The Brumadinho DVI relied on the robust odontological AM database. Failure to apply odontology in Brumadinho was related to dismemberment or lack of dental samples for analysis (disrupted postmortem) rather than the quality of odontological documentation. An important factor indicated in one study for using PLASSdata software (employed for the first time in a DVI in Brazil) is the need for the teams’ training and familiarity with that method [19].

The use of combined identification methods accounted for 16.0% of the primary identifications in Brumadinho. Combined methods may be applied for DVI even though forensic teams aim for swifter primary identification methods. It is possible for such teams to collaborate, working at different locations or laboratories; thus, using combined as in Brumadinho is not unprecedented [28, 32].

A detailed investigation into the cause of death is important for a police inquiry; the medical examiner is responsible for recreating the dynamics of trauma using necroscopic findings [6, 16]. In Brumadinho, an autopsy was performed for every case. However, some disasters may require swifter, more optimised necroscopic examinations, employing virtopsy or external examinations in every case and leaving necroscopic examination only for eligible cases [6, 7, 14, 28, 33].

We observed that PTI was the predominant cause of death, accounting for 95.2% of cases. The great mechano-kinetic energy of the tailings and their destructive action on the victims’ bodies is the causal factor that best explains this finding [2]. Computerised tomography and three-dimensional reconstruction have also been used to determine the causes of death with such disasters [14].

In 29 cases, we identified other causes of death—isolated or in association with traumatic injury, notably asphyxia. Those cases involved whole bodies or parts of bodies including the head and torso that underwent autopsy up to the 42nd day after the disaster. Those findings helped illuminate causes of death other than PTI and had the shortest time of identification, and the cases presented essential viscera for analysis. Diagnosing asphyxia is important because it is a cause of death that qualifies as homicide according to the criminal legislation of Brazil (subsections I and II, § 2nd of Article 121 of the Penal Code).

The destructive force of the tailings extended beyond the limits of the mining plant, resulting in 21 community deaths, with a 20% dismemberment rate. The dismemberment among workers was three- to four-fold that of the community victims, which emphasises the destruction and higher mechano-kinetic energy of the tailings within the plant (Supplementary Figure 1). The dismemberment among VALE SA and third-party employees was almost equal. This finding supports those of reports in the specialist literature, which indicate the tendency to outsource activities in the mining sector—notably those with higher occupational hazards [34–36].

## Conclusion

Every disaster and the responses by specialist teams have particular features that obstruct efforts to define parameters or means of comparison. In response to the Brumadinho disaster, some points on medical and forensic management are noteworthy. The degree of dismemberment and extension of the search period and locations were responsible for two well-defined stages in the disaster.

In the first stage, the prioritisation of friction ridge analysis proved effective: it was responsible for most of the total primary identifications; it allowed a swift response and cost-effective DVI. The need to extend the search period resulted in registering cases in various stages of decomposition. The dismemberment resulting from the destructive force of the tailings on the victims’ bodies added to the decomposition and increased the complexity in identifying cases.

During the second stage of the DVI, DNA analysis was responsible for most of the identifications. Among primary identifications, it accounted for the second-highest number of cases; with re-identification, it was the most-used method. Employing other methods allowed for swift identification of victims, avoiding excessive use of DNA analysis.

Identification is the goal with DVIs. With Brumadinho, autopsies were conducted on every case. The autopsies used all available professional and technological resources to make an exhaustive examination of causes of death; they confirmed the mechanisms of death and whether the deaths qualified as homicide under Brazilian criminal legislation.

We observed a greater number of deaths and incidences of dismemberment among the mining employees than with community residents. We also found a higher proportion of third-party than VALE SA employees. In addition to helping understand the dynamics of this particular tragedy, our findings contribute to research on occupational hazards for mine workers.

With disasters, the highest mortality occurs in developing countries. However, fewer than 1% of studies on disasters have taken place in such countries; fewer than 25% of those authors were native to those countries. The present study aimed to close that gap.

## Supplementary Material

Supplemental Material
