# Supplementary material for: Identification of victims of the collapse of a mine tailing dam in Brumadinho
Source: Forensic Sci Res. 2023 Feb 12;7(4):580–9. doi: 10.1080/20961790.2022.2113623 (PMC9930756; doi:10.1080/20961790.2022.2113623)
Supplement: Supplemental Material [file TFSR_A_2113623_SM1137.docx]

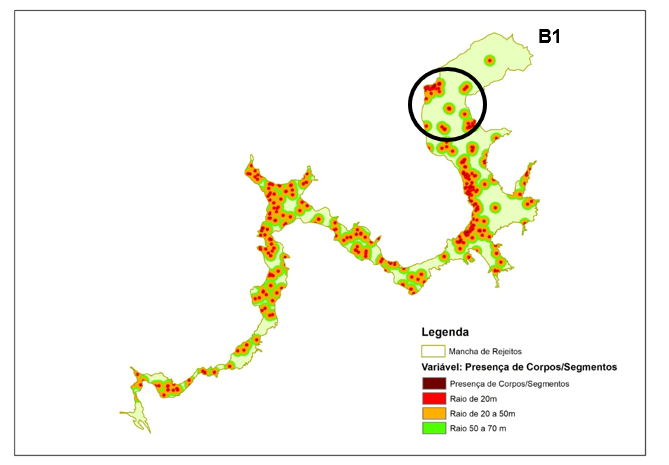
**Supplementary figure 1.** Dispersion of bodies and body segments in the

**Supplementary Table 1.** Identification method according to body part found.

| Body part | Anthropology | DNA | Associated methods | Odontology | Papiloscopy | *P*-value |
| --- | --- | --- | --- | --- | --- | --- |
| Whole body | 1 (20) ^a^ | 1 (0.3) ^a^ | 7 (41.2) | 4 (12.9) | 70 (36.3) | <0.001 |
| Inc. head, inc. torso, comp. SL. | - | 1 (0.3) | 1 (5.9) | 3 (9.7) | 19 (9.8) | 0.990 |
| Comp. head. inc. torso. inc. SL. | - | - | - | 2 (6.5) | 1 (0.5) | 0.999 |
| Inc. torso, comp. SL | - | 2 (0.6) | - | - | 4 (2.1) | 0.481 |
| Inc. torso, inc. IL | - | 38 (10.6) | - | - | - | - |
| Inc. head, inc. torso, inc. SL, inc. IL. | - | 2 (0.6) | - | 1 (3.2) | - | 0.218 |
| Comp. head, comp. torso, inc. SL, inc. IL. | - | - | - | 2 (6.5) | - | - |
| Inc. head, comp. torso, inc. SL, inc. IL. | - | - | - | 2 (6.5) | - | - |
| Comp. torso. inc. SL. inc. IL. | - | 1 (0.3) | - | - | 1 (0.5) | 0.982 |
| Inc. torso, comp. SL, inc. IL. | - | - | - | - | 5 (2.6) | - |
| Comp. torso. comp. SL. inc. IL. | - | - | - | - | 2 (1) | - |
| Comp. head, comp. torso, comp. SL, comp. IL, body almost whole | - | - | 3 (17.6) | - | 14 (7.3) | 0.999 |
| Inc. torso, comp. MI | 1 (20) | 38 (10.6) | - | - | - | 0.512 |
| Comp. torso. inc. SL. comp. IL. | - | - | - | 1 (3.2) | - | - |
| Inc. torso, comp. SL, comp. IL. | - | - | 2 (11.8) | - | 5 (2.6) | 0.999 |
| Comp. torso. comp. SL. comp. IL. | 1 (20) | - | - | 1 (3.2) | 22 (11.4) | 0.182 |
| Inc. torso, inc. SL | - | 10 (2.8) | - | - | - | - |
| Inc. head. inc. torso. | - | 1 (0.3) | 1 (5.9) | 1 (3.2) | - | 0.999 |
| Comp. head. inc. torso. | - | - | - | - | 1 (0.5) | - |
| Comp. head. comp. torso. | - | - | - | - | 1 (0.5) | - |
| Comp. head. inc. SL. | - | - | - | 1 (3.2) | 2 (1.0) | 0.104 |
| Comp. head. inc. torso. comp. SL. | - | 1 (0.3) | - | - | 11 (5.7) | 0.997 |
| Comp. head. comp. torso. comp. SL. | - | - | - | 1 (3.2) | 4 (2.1) | 0.999 |
| Inc. head, inc. torso, inc. SL. | 1 (20) ^a b^ | 3 (0.8) ^a^ | 1 (5.9) | 3 (9.7) | 1 (0.5) ^b^ | **<0.001** |
| Inc. head, comp. torso, comp. SL. | - | - | 1 (5.9) | - | 2 (1.0) | 0.999 |
| Only biological tissue | - | 75 (21.0) | - | - | - | - |
| Only inc. SL. | - | 21 (5.9) | - | - | 16 (8.3) | 0.282 |
| Only comp. SL. | - | 6 (1.7) | - | - | 12 (6.2) | 0.997 |
| Only inc. IL. | 1 (20) | 100 (28) | - | - | - | 0.694 |
| Only comp. IL. | - | 16 (4.5) | - | - | - | - |
| Only complete torso | - | 30 (8.4) | - | - | - | - |
| Only incomplete head | - | 10 (2.8) | 1 (5.9) | 7 (22.6) | - | 0.998 |
| Only complete head | - | 1 (0.3) | - | 2 (6.5) | - | 0.999 |
| **Total** | **5** | **357** | **17** | **31** | **193** | **-** |

The p-values refer to the binary logistic model. Overwritten letters indicate significant differences. comp. = complete; inc. = incomplete; SL = superior limb; IL = inferior limb.
